# Assisted reproductive technology outcomes and gene expression in unexplained infertility patients

**DOI:** 10.3389/fcell.2023.1217808

**Published:** 2023-07-28

**Authors:** Brigita Vaigauskaitė-Mažeikienė, Raminta Baušytė, Elvina Valatkaitė, Rūta Maželytė, Edita Kazėnaitė, Diana Ramašauskaitė, Rūta Navakauskienė

**Affiliations:** ^1^ Department of Molecular Cell Biology, Institute of Biochemistry, Life Sciences Center, Vilnius University, Vilnius, Lithuania; ^2^ Centre of Obstetrics and Gynaecology of the Institute of Clinical Medicine, Faculty of Medicine, Vilnius University, Vilnius, Lithuania; ^3^ Faculty of Medicine, Vilnius University Hospital Santaros Klinikos, Vilnius University, Vilnius, Lithuania

**Keywords:** unexplained infertility, assisted reproductive technology, gene expression, endometrium, follicular fluid (FF)

## Abstract

**Background:** Unexplained infertility (UI) can be a frustrating and challenging diagnosis for doctors and couples as it can be difficult to understand why they are unable to conceive despite increasing diagnostic tools. Assisted reproductive technology (ART) procedures have been successfully applied to many couples aiming to overcome UI. However, they can be not only expensive but also require multiple cycles to achieve a successful pregnancy. The endometrium and the follicular fluid have been investigated as target tissues not only to determine the cause of UI but also to increase conception rates.

**Results:** In this study, we analyzed the outcomes of ART in 223 UI couples and gene expression associated with DNA modification, cell death, immune response and senescence (*TET1, TET2, BCL2, BAK1, HMGA2, IL-6, IL-8*) in infertile women’s endometrium and follicular fluid. We found significant differences in women who successfully got pregnant compared to women unable to conceive depending on age, duration of infertility, number of retrieved oocytes, zygotes, transferred embryos. Further, the expression of genes *BAK1* (pro-apoptotic)*, TET2* (associated with epigenetic DNA modification) and *IL-6* (associated with immune responses) were significantly higher in the endometrium of women who successfully got pregnant.

**Conclusion:** Younger parental age couples showed higher ART success rates, shorter duration of infertility, higher number of retrieved oocytes, zygotes and transferred embryos. The gene expression analysis revealed significant changes in the endometrium depending on genes associated with cell death and immune response which were upregulated in females with diagnosed unexplained infertility.

## Introduction

Infertility is a disease of the reproductive system defined by the failure to achieve a clinical pregnancy after 12 months or more of regular unprotected sexual intercourse. *Unexplained infertility (UI)* term exists longer than 50 years with the definition of couples who both have normal genito-urinary anatomy, normal semen quality and adequate sexual intercourse (Zegers-Hochschild et al., 2017). However, when defining the term “unexplained”, emphasis is laid on the quality of diagnostic tests and tools (Sadeghi, 2015). According to different authors (Evers, 2002; Gelbaya et al., 2014), UI varies approximately from 10 to 30 percent in all infertile couples, although discussions among clinicians with reference to “unexplained” actually as “undiagnosed” are present (Gleicher and Barad, 2006; Sadeghi, 2015). Other tempting and possible causes of infertility such as minor ovarian and testicular dysfunctions, the quality of sperm and oocyte, endometrial receptivity, implantation failures, immune factors dysfunction are still underway to daily clinical practice. Some studies suggest that up to 15% of these young couples (less than 30 years old) will conceive over a 1-year period and up to 30% over the second year, therefore expectant management is suggested (Isaksson and Tiitinen, 2004). On the other hand, it has been highlighted that women over 35 years of age are diagnosed with unexplained infertility twice as often as younger women (less than 30 years old) (Maheshwari et al., 2008). Even though up to six cycles of ovarian stimulation or/and intrauterine insemination can be offered to couples with UI by many fertility organization guidelines, yet *in vitro* fertilization procedure (IVF) is considered an effective first-line treatment in UI patients (Pandian et al., 2001; Buckett and Sierra, 2019). In the couples who received IVF, subtle changes were observed in oocyte fertilization rates indicating that the pathogenesis of UI may lie in the fertilization process itself which remains indistinct for diagnosis (Hull, 1994). Also, more attention has been brought to a search of molecular mechanisms underlying UI. Some studies indicate that the endometrium is a key tissue in the UI pathogenesis considering, for instance, the reduced expression of Foxp3 (a master regulator of Treg cell differentiation) decreases the endometrial Treg cell population resulting in implantation failure (Jasper et al., 2006). Other researchers indicate that UI is associated with a compromised endometrial receptivity during the implantation window in mechanisms such as the reduction of αVβ3 integrin in the endometrium (Elnaggar et al., 2017), overexpression of endometrial estrogen receptor-alpha (ER-α) in the mid-luteal phase (Dorostghoal et al., 2018). Furthermore, follicular fluid (FF) components have been investigated to discover some new insights. Some authors showed that the lipid compound in FF could affect oocyte development: not only higher amounts of phospholipid but also phospholipid/apoA-I ratio in FF were associated with poor oocyte fertilization rates (Fayezi et al., 2014). Also, some studies indicate that the reduction of some components (triglycerides, lactate, alkaline phosphatase and lactate dehydrogenase) is negatively associated with follicle size indicating impaired oocyte maturation (Nandi et al., 2007). In addition, increased triacylglycerol levels with a lower amount of monoacylglycerols, phospholipids and sphingolipids were found in the FF of UI population (Batushansky et al., 2020).

Gene expression in either the endometrium or the follicular fluid has been investigated to determine underlying molecular variations partially contributing to UI. The study conducted by Altmäe et al. detected several important genes (such as the mucin-associated peptide *TFF3*; metalloproteinases *MMP8*, *MMP10*, *MMP26*; chemokines *SCBG3A1*, *FAM3D*, *FAM3B*; the integrin-binding protein *COL16A1*), *etc.*) which were dysregulated in infertile women with UI (Altmäe et al., 2009). It shows the occurrence of subtle changes in the immune responses, signal transduction, binding, transport, lipid metabolism and extracellular matrix component at the time of embryo implantation in women with unexplained infertility compared to fertile women. Furthermore, complement-coagulation cascades, morphine addiction pathway, and PI3K-Akt signalling pathway were determined to be differentially expressed in UI patients which reveals the complexity of UI diagnosis (Keleş et al., 2022). It is known that TET family genes are associated with DNA demethylation process and its decrease was noticed in infertile endometriosis patients (Adamczyk et al., 2022a). *BCL2* and *BAK1* genes are cell death regulators and sometimes irregular patterns of the expression of these genes could reveal some underlying issues, for example, cancer (Witek et al., 2016). In this case, we wanted to see if there are any changes in expression patterns in unexplained infertility patients. Further, some evidence suggests that the decrease of high mobility group AT-hook 2 (*HMGA2*) expression reduces stem cell frequency and function (Nishino et al., 2008). These major pathways are responsible in ensuring that all cellular processes are being regulated normally and are not being altered like in the cases of diseases. For example, some pro-inflammatory cytokines (IL-6, IL-8) are related to inflammation, which may be responsible not only for failed IVF, but also could be associated with unexplained infertility (An et al., 2015; Fortin et al., 2019). Our goal was to search for unconventional genes related to this topic and see if there is a significant difference between infertile patients who conceived successfully and failed to conceive as well as investigate possible associations with infertility and pregnancy outcome. When analyzing follicular fluid gene expression, some studies suggest that an imbalance between pro-inflammatory and anti-inflammatory mediators could be the underlying reason in the failure of conception after ART cycles (Fortin et al., 2019). Unfortunately, there is a lack of research of the gene expression in follicular fluid in humans concerning oocyte maturation, fertilization, aging processes.

While focusing on long term outcomes of UI couples, the bright side is that most UI couples were able to achieve a live birth either spontaneously or when treated (Pandian et al., 2001; Vaughan et al., 2022). An important conclusion was drawn that couples who do not delay the treatment have greater odds to achieve more than one live birth.

In the current study, we analyzed the reproductive outcomes of UI couples who received ART and performed the subgroup analysis addressing the conceived and failed to conceive populations. Moreover, to carry out the gene expression analysis in the endometrial and follicular fluid tissues, selected women from these populations. Our study investigated seven genes (*TET1, TET2, BCL2, BAK1, HMGA2, IL-6, IL-8*) associated with the DNA modification, cell death, immune response and senescence. The findings of this study not only could help understand the unexplained infertility diagnosis but also improve the conception outcomes when treating these couples.

### Primary and secondary outcomes

Our primary outcome measure in this study was the cumulative overall ongoing pregnancy rate (OPR). Our secondary outcome measure was the conception predicting factors.

## Materials and methods

**FIGURE 1:**
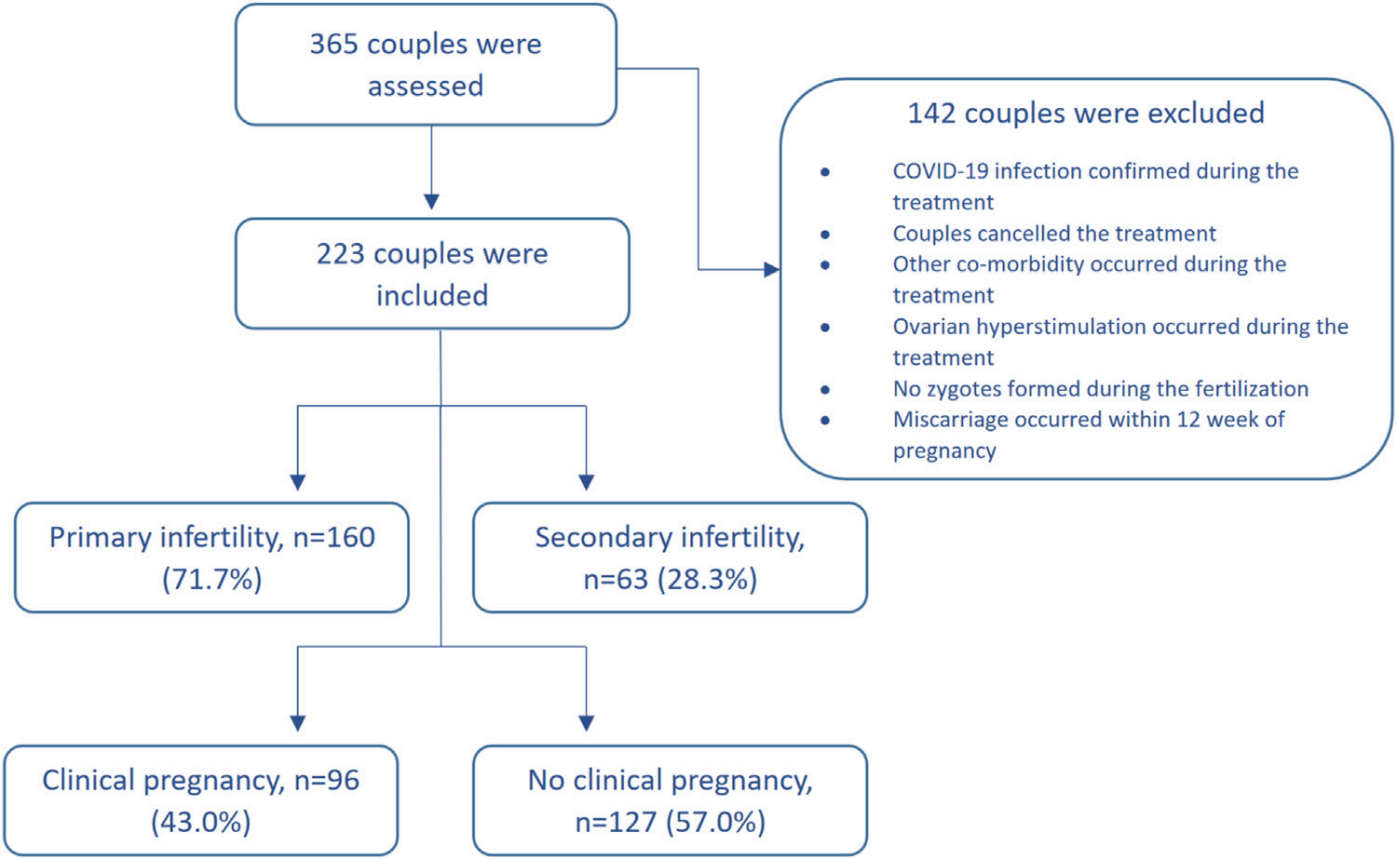
Study flowchart.

We have conducted an interventional prospective cohort study. The patients with unexplained infertility who received ART and were treated in Santaros Fertility Center of Vilnius University Hospital Santaros Klinikos were enrolled for the study from 1 January 2020 to 30 March 2021 (until the COVID-19 lockdown). The inclusion criteria were as follows: 1) age of women at the time of enrollment was 25–40 years, 2) average duration of infertility at least 2 years, 3) unexplained infertility diagnosis was confirmed after laboratory and instrumental investigation (testing failed to reveal any abnormality), 4) informed consent of all the subjects was received. The exclusion criteria were as follows: 1) oncological disease was confirmed for a woman during the last 3 years, 2) other infertility causes, except unexplained infertility, were confirmed, 3) women who were addicted to alcohol or other substances, 4) uncontrolled endocrine or other medical conditions, such as prolactinemia or thyroid diseases, 5) COVID-19 infection was confirmed during the treatment. The flow chart of the study population is presented in Figure 1.

All women received endometrial scratching procedure without the dilatation of the cervix, using a pipelle catheter during the natural luteal cycle phase (approximately 7–9 days before the menstrual cycle and the start of ovarian stimulation). The hormones (estradiol, luteinizing hormone and progesterone) were investigated during the second-third day of the cycle before starting ART. All women underwent controlled ovarian stimulation by the GnRH antagonist protocol or short GnRH agonist protocol. The ovarian response was assessed by transvaginal ultrasound. Human chorionic gonadotropin was administered to induce the final oocyte maturation 36 h before oocyte retrieval. Oocyte retrieval was performed under general anesthesia using transvaginal aspiration with 16–17-gauge needles under ultrasonography guidance. Oocytes were extracted by an embryologist, and the residual FF was pooled and spun down to collect FF and discard blood and granulosa cells. Fertilization was accomplished by IVF or intracytoplasmic sperm injection. Day 3 or day 5 embryos were transferred according to the age of the woman, anamnesis of infertility, the embryo morphologic grading. For the luteal phase support, a total of 600 mg intravaginal progesterone was prescribed. A human chorionic gonadotropin (HCG) pregnancy test was carried out 14 days after the oocyte retrieval procedure. An ongoing pregnancy was defined with each pregnancy showing a positive heartbeat of the fetus at ultrasound after 12 weeks of gestation.

### Collection of follicular fluid and endometrial tissue

Endometrium samples were collected during the scratching procedure. Follicular fluid containing heterogenic population was collected at the time of oocyte aspiration without flushing (36 h after human chorionic gonadotropin (6500 IU) trigger administration) into sterile tubes. Follicular fluid samples used for analysis were macroscopically clear and not contaminated with blood. Once collected, follicular fluid was transferred into 50 mL tubes and centrifuged at 500× g for 10 min, supernatant removed and 20 mL of 1× red blood cell (RBC) lysis buffer (10×, 155 mM NH_4_Cl (Sigma-Aldrich, St. Louis, MO, United States), 12 mM NaHCO_3_ (Sigma-Aldrich, St. Louis, MO, United States), 0.1 mM EDTA (Sigma-Aldrich, St. Louis, MO, United States), pH 7.3) was added to the pellet. Further, the samples were incubated for 5 min at room temperature and centrifuged at 500× g for 10 min. After centrifugation, the isolated heterogenic cell pellet was mixed with DNA/RNA lysis buffer (Zymo research, Irvine, CA, United States) and incubated at room temperature up to 10 min. After this, RNA isolation followed as described below.

### RNA isolation and gene expression analysis

For the total RNA extraction from follicular fluid cells, the Quick-DNA/RNA Miniprep Kit (Zymo Research, CA, United States) was used in line with manufacturer’s recommendations. The RNA isolation from the endometrium tissue required an additional step prior to using the kit. A piece of the tissue was first submerged in liquid nitrogen and ground to a powder using a mortar and pestle. Then the powder was mixed well with DNA/RNA lysis buffer and the lysate was transferred to a Zymo-Spin™ Column. The following isolation steps were carried out according to manufacturer’s recommendations. After this, cDNA was synthesized with the LunaScript^®^ RT SuperMix Kit (New England Biolabs, Ipswich, MA, United States) following manufacturer’s guidelines. cDNA was then amplified using the RT-qPCR with Luna^®^ universal qPCR Master Mix (New England Biolabs, Ipswich, MA, United States) and the Rotor-Gene 6000 Real-time Analyzer (Corbett Life Science, QIAGEN, Hilden, Germany) with experimental conditions as follows: 95°C 1 min, 95°C 15 s, 60°C 30 s (40 cycles). Primer sequences used in RT-qPCR analysis are listed in Table 1. The acquired data from RT-qPCR was further analyzed using the ΔΔCt method, mRNA expression was normalized using *GAPDH* gene expression and therefore the relative gene expression was calculated.

**TABLE 1:** Primer sequences used in gene expression analysis.

Name	Primer sequence	Product size, bp
*TET1*	F: TTC​GTC​ACT​GCC​AAC​CTT​AG	149
	R: ATG​CCT​CTT​TCA​CTG​GGT​G	
*TET2*	F: CCC​TTC​TCC​GAT​GCT​TTC​TG	136
	R: TGG​GTT​ATG​CTT​GAG​GTG​TTC	
*BCL2*	F: CGG​AGG​CTG​GGA​TGC​CTT​TG	166
	R: TTT​GGG​GCA​GGC​ATG​TTG​AC	
*BAK1*	F: TCA​TCG​GGG​ACG​ACA​TCA​AC	120
	R: CAA​ACA​GGC​TGG​TGG​CAA​TC	
*IL-6*	F: ACA​GCC​ACT​CAC​CTC​TTC​AG	168
	R: CCA​TCT​TTT​TCA​GCC​ATC​TTT	
*IL-8*	F: CAT​ACT​CCA​AAC​CTT​TCC​ACC​CC	175
	R: TCA​GCC​CTC​TTC​AAA​AAC​TTC​TCC​A	
*HMGA2*	F: CCC​AAA​GGC​AGC​AAA​AAC​AA	81
	R: GCC​TCT​TGG​CCG​TTT​TTC​TC	
*GAPDH*	F: GTG​AAC​CAT​GAG​AAG​TAT​GAC​AAC	123
	R: CAT​GAG​TCC​TTC​CAC​GAT​ACC	

## Results

Two hundred twenty-three couples were enrolled in the study of whom 127 (57%) were unable to conceive (Group A) while 96 (43%) couples successfully got pregnant (Group B). The characteristics of the total cohort and study groups are presented in Table 2.

**TABLE 2:** Patient characteristics for the total cohort and study groups.

Characteristics	Study cohort (*n = 223*)	Group A (*n* = *127*)	Group B (*n* = 96)	*p* values
Women age, in years	33.4 ± 3.7	34.1 ± 3.8	32.6 ± 3.6 years	*p* **= 0.006**
Mean ± SD				
Men age, in years	35.3 ± 5.1	36.0 ± 5.1	34.5 ± 5.2	*p* **= 0.03**
Mean ± SD				
Women BMI, kg/m2	22.4 ± 3.7	22.3 ± 3.2	22.8 ± 4.3	*p* = 0.99
Mean ± SD				
Smoking male, number	58	28	30	*p* = 0.12
The duration on infertility, in years	4.8 ± 2.9	5.2 ± 3.2	4.4 ± 2.5	***p* = 0.01**
Mean ± SD				
Type on infertility, number	160	88	72	*p* = 0.35
Primary				
Secondary	63	39	24	
Stimulation protocol, number	176	125	51	*p* = 0.29
Antagonist				
Agonist	47	10	37	
Retrieved oocytes, number	11.3 ± 9.0	6.7 ± 9.2	12.3 ± 9.7	***p* < 0.001**
Mean ± SD				
Zygotes, number	5.7 ± 5.5	3.4 ± 5.3	7.0 ± 6.8	***p* < 0.001**
Mean ± SD				
Total transferred embryos: 44.2% of day3 embryos; 55.8% of day5 embryos; number	1.3 ± 1.1	1.0 ± 1.0	2.0 ± 1.0	***p* = 0.016**
Mean ± SD				

*p* values marked in bold indicate statistically significant results.

**FIGURE 2:**
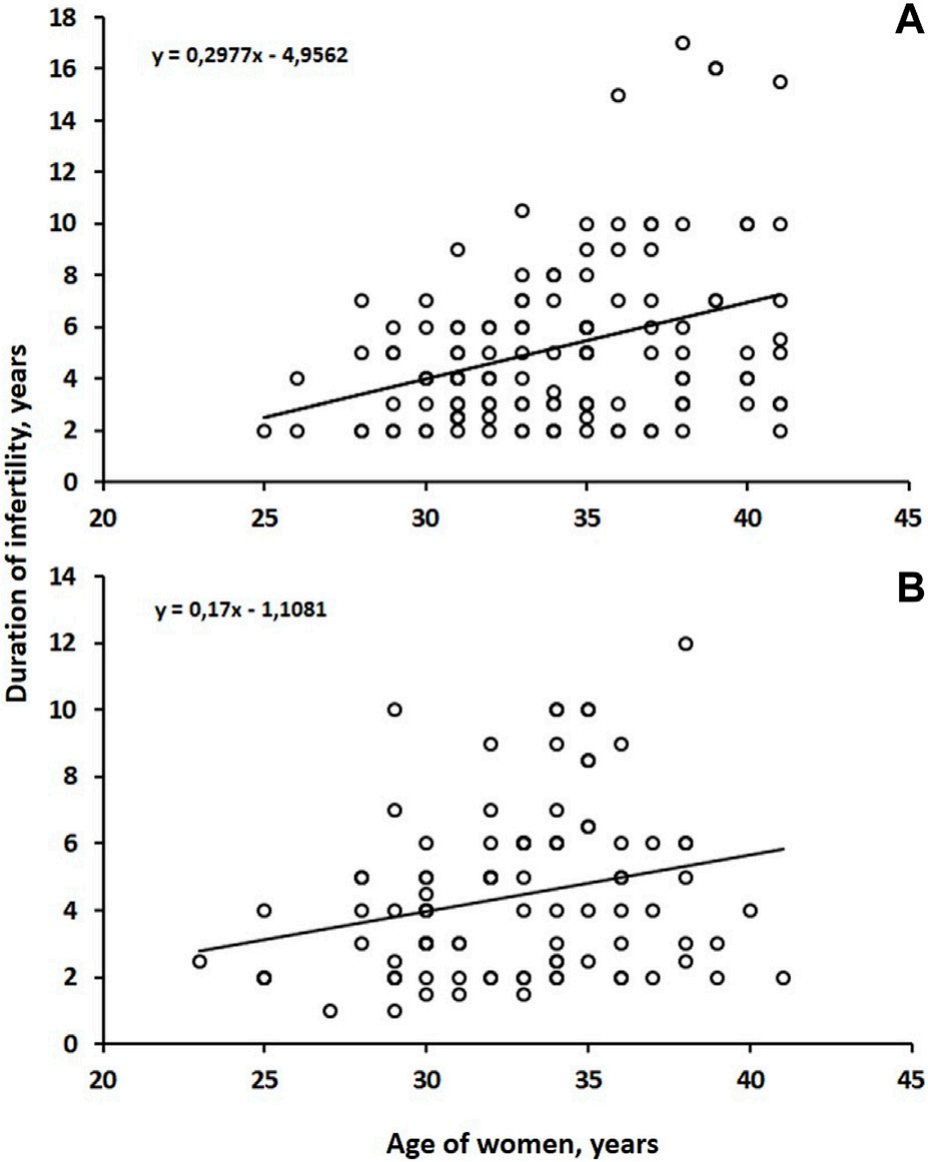
Correlations between the age and duration of infertility in both groups.

A statistically positive correlation was found between the age of both groups (Group B r = 0.26; *p* = 0.01) (Group A r = 0.30; *p* = 0.001) and the duration of infertility (Figure 2).

When analyzing the spermogram indicator values of the participating men, the sperm of Group B men had statistically higher values of sperm count, however, the values of live sperm, sperm motility A (rapid progressive motility) and sperm motility B (slow or sluggish progressive motility) were higher in Group A (Table 3).

**TABLE 3:** Mean, minimum (Min), maximum (Max) values and standard deviations of spermogram indicators of men from both groups.

Spermogram indicator	Group	Mean	Standard deviation	Minimal value	Maximal value	*p*-value
Sperm count, mln./ml	A	77.5	59.4	0.0	285.0	**0.01**
	B	97.9	61.7	3.0	322.0	
Live sperms, %	A	52.1	18.7	10.0	150.0	0.25
	B	49.2	20.3	10.0	160.0	
Motility A, number	A	24.5	26.1	0.0	232.0	**0.01**
	B	18.1	16.7	0.0	70.0	
Motility B, number	A	20.6	10.4	0.0	61.0	**0.02**
	B	17.5	12.5	0.0	66.0	

*p* values marked in bold indicate statistically significant results.

To evaluate the correlations of the conception success with endometrium thickness, the influence of seven different female hormones, the number of retrieved oocytes, zygotes and transferred embryos, the non-parametric values ​​of the Spearman’s correlation coefficients were calculated. Our analysis revealed a significant conception success correlation with estradiol and progesterone hormones, the number of retrieved oocytes, zygotes and the number of the total transferred embryos (in all cases Spearman; *p* < 0.05), whereas no significant correlation was found between the successful conception and endometrial thickness and the remaining female hormones (in all cases Spearman; *p* > 0.05) (Table 4).

**TABLE 4:** Pregnancy success correlation with endometrial thickness, values of different female sex hormones concentration, number of retrieved oocytes, zygotes, and number of transferred embryos.

Characteristics	Success in conception	
	r	p
Endometrium thickness	0.13	0.30
Luteinizing hormone	−0.04	0.47
Follicle-stimulating hormone	0.07	0.30
Estradiol	−0.14	**0.04**
Progesterone	0.15	**0.03**
Prolactin	−0.006	0.92
Anti-Mullerian hormone	0.13	0.06
Thyroid stimulating hormone	0.05	0.49
Retrieved oocytes	0.34	**< 0.001**
Zygotes	0.34	**< 0.001**
Total transferred embryos	0.31	**< 0.001**
Transferred blastocysts	0.28	**< 0.001**

p values marked in bold indicate statistically significant results.

A detailed correlational analysis showed very a weak negative significant statistical correlation between the successful conception and estradiol hormone (on day 2/3 when the ovarian stimulation was started), indicating that as the concentration of the hormone increased, the probability of conception reliably decreased (r = −0.14; *p* = 0.04) (Table 4). The opposite result was obtained between the successful conception and hormone progesterone, as a very weak positive statistical correlation was formed between these criteria (r = 0.15; *p* = 0.03). This suggested that the increasing concentration of hormone progesterone significantly increases the chances of conception (Table 4). It is important to recall that all women have started an IVF cycle and ovarian stimuliation when their progesterone levels were in normal range at day 2/3 (≦ 1.0 ng/mL).

During further statistical analysis, weak positive correlations were observed between the successful conception and the number of retrieved oocytes (r = 0.34; *p* < 0.001); number of zygotes (r = 0.34; *p* < 0.001) and the number of the total transferred embryos (r = 0.31; *p* < 0.001). A very weak positive (r = 0.28; *p* < 0.001) significant statistical correlation was found between the successful conception and the number of transferred blastocysts (Table 5). Statistically significant relations between the age of women of Group A and the duration of infertility and an anti-Mullerian hormone were found implying that the increased age is also related to a longer duration of infertility and a decreased chance to conceive.

**TABLE 5:** Association between unsuccessfully and successfully conceived women age and duration of infertility, number of retrieved oocytes, number of fertilized oocytes and AMH hormone.

Characteristics	Women age			
	Group A women​		Group B women	
	r	p	r	p
Duration of infertility	0.30	**0.001**	0.26	**0.01**
Number of retrieved oocytes	0.27	0.08	−0.14	0.23
Number of zygotes	0.12	0.17	−0.17	0.10
Anti-Mullerian hormone	−0.32	**< 0.001**	−0.18	0.09

*p* values marked in bold indicate statistically significant results.

With the help of RT-qPCR, we tested seven genes associated with the epigenetic DNA modification (*TET1*, *TET2*), senescence (*HMGA2*), apoptosis (*BCL2, BAK1*) and the immune response (*IL-6, IL-8*) in the endometrium tissue (End) and the follicular fluid (FF). Nevertheless, our interest was set not only within these groups, but also between Group A and Group B under our analysis. To conduct this analysis, we selected 10 samples to represent End group (Group A *n* = 5; Group B *n* = 5) and 10 samples to represent FF group (Group A *n* = 5; Group B *n* = 5).

**FIGURE 3:**
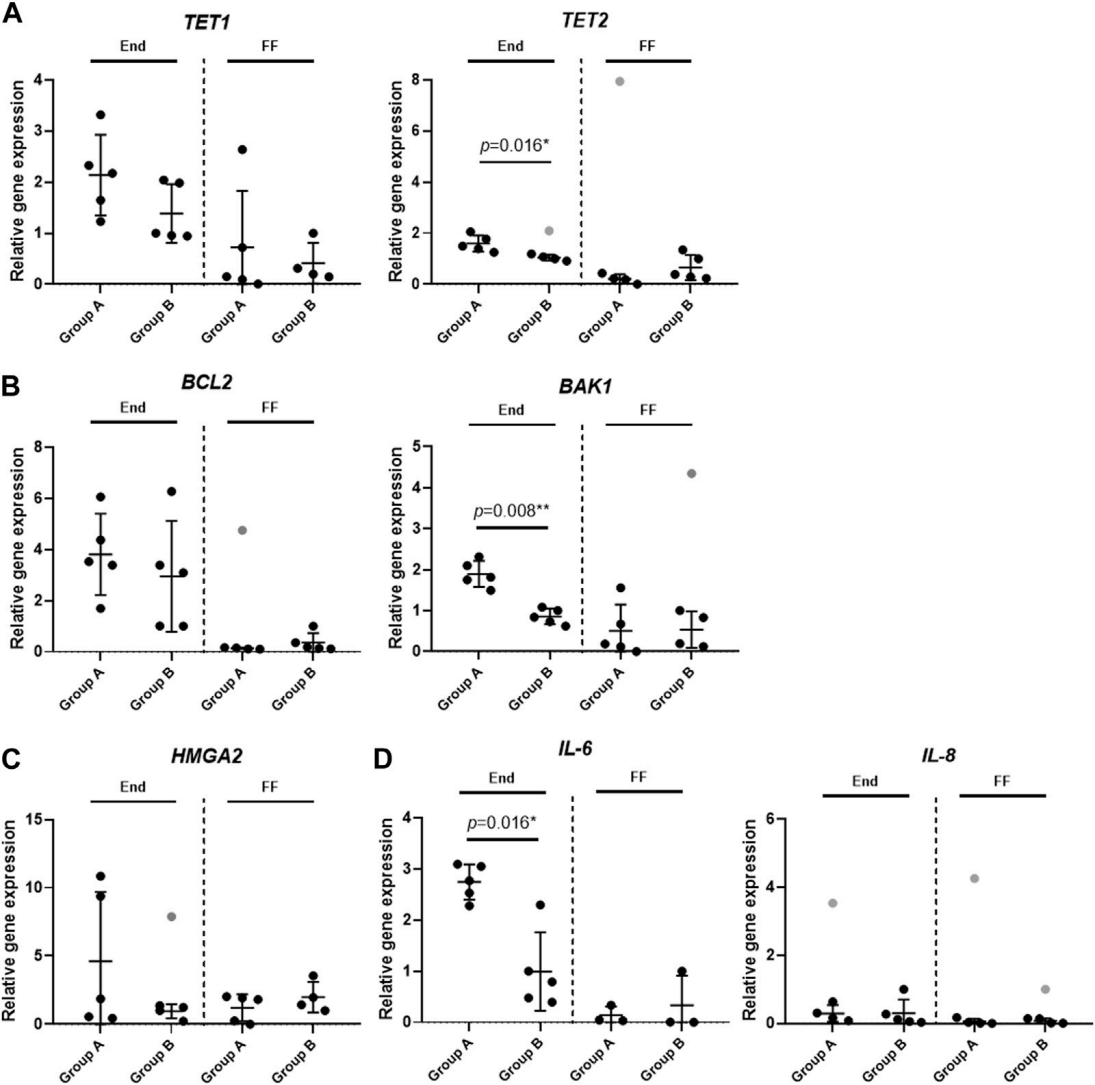
Analysis of gene expression profiles in endometrium and follicular fluid. Gene expression was analyzed in Group A (unexplained infertility patients; *n* = 5) and Group B (fertile patients; *n* = 5) using RT-qPCR. **(A)** Epigenetic DNA modification-related genes (*TET1, TET2*) expression analysis. **(B)** Gene expression analysis of cell death associated genes (*BCL2, BAK1*). **(C)** Senescence associated *HMGA2* gene expression analysis. **(D)** Gene analysis of genes related to immune response (*IL-6, IL-8*). Relative gene expression analysis was calculated using ΔΔCt method and *GAPDH* was used as a reference gene. Data are presented as mean ± SD; grey data points depict outliers as determined by ROUT (Q = 2%). Statistical significance was determined with Mann–Whitney *U* test and significance was set to *p ≤ 0.05; **p ≤ 0.01. Unmarked data points show no significance.

The *TET1* and *TET2* gene expression involved in the DNA demethylation process is seen to be slightly upregulated in End group compared to FF group. In the case of *TET1* gene expression profile, no significant differences were detected between Group A and Group B, however, *TET2* gene was significantly upregulated in Group A in End group compared to Group B (Figure 3A). Similar tendency can be observed in the anti-apoptotic gene *BCL2* expression profile where the expression of this gene was very similar between Group A and Group B in both End and FF groups (Figure 3B). On the other hand, we detected that the expression of pro-apoptotic gene *BAK1* was significantly higher in Group A End group compared to Group B (Figure 3B). However, no such difference was observed in FF group. Senescence associated *HMGA2* gene expression showed a slight tendency to be upregulated in Group B FF group, however, in End group, the expression remained the same between Group A and Group B (Figure 3C). Further genes under our focus were *IL-6* and *IL-8* associated with immune responses. The *IL-6* gene expression analysis revealed that in the endometrium of Group A the expression of this gene increased significantly compared to Group B (Figure 3D). On the contrary, FF group showed no significant changes. Moreover, *IL-8* expression remained at low levels in all of the groups including Group A and Group B.

## Discussion

Unexplained infertility diagnosis remains challenging both for the patients and clinicians. Not only the patients delay the infertility treatment due to the knowledge to be a “healthy” couple, but also the lack of understanding what does not function in the body triggers the couples. From the clinicians’ perspective, understanding the pathogenesis of the diagnosis is the key to the proper evaluation and treatment the disease. Our study investigates the clinical data and gene expression of the UI patients. The findings of our research help to understand the clinical and molecular view of the UI and indicates the direction for further investigation.

Age is widely known as the key element not only in terms of the ovarian reserve and function but also possible conception. It is estimated that female fertility declines significantly after the age of 35 leaving them with a chance to conceive spontaneously of around 66% and a dramatic drop later in years (Delbaere et al., 2020). What is more, according to some studies, women of the same age with 2 years of primary unexplained infertility have even lower chances to conceive (Chua et al., 2020). Interestingly, some authors even raise an idea that age-related infertility and UI diagnosis are contradictory and should not be assimilated (Somigliana et al., 2016). Despite the diagnosis, older women have naturally lower number of oocytes which leads to a lower amount of embryos in IVF cycles and a decreasing implantation rate (Spandorfer et al., 1998). A very important study revealed that embryo implantation rates in IVF cycles remain constant until the age of 35 and later decrease by 2.77% per year (Spandorfer et al., 2000). This could be associated with such factors as the decreased uterine receptivity, older paternal age, embryo aneuploidy, *etc.* Our study found that younger female age is a statistically reliable factor in prognosing the conception in UI patients after IVF. Also, the male age was significantly lower in the clinical pregnancy group. We believe that it should be emphasized for the UI couples that the treatment delay (especially when considering IVF) could negatively affect their conception rate.

A fertilization method is always a matter of question in performing the ART cycle for UI patients. The purpose of performing the intracytoplasmic sperm injection (ICSI) procedure prior to a conventional *in vitro* fertilization (IVF) in UI couples was to increase fertilization rates as a possible reason of infertility (Practice Committees of ASRM and SART, 2020). Several studies have been conducted to estimate whether the conception rates were higher in ICSI groups when treating UI patients. Unfortunately, most of the authors did not find any higher clinical pregnancy rates associated with ICSI although higher fertilization rates were observed (Ruiz et al., 1997; Foong et al., 2006). Similarly, our study showed that ICSI for UI couples did not improve clinical pregnancy rates. These findings suggest that ICSI should not be performed as a “rescue” procedure for UI patients especially if it is the first ART cycle.

A growing number of recent research studies suggest that the impaired quality of oocyte could be the key factor in UI patients’ conception likely to lead to a poor-quality embryo formation (Tejera et al., 2005). However, in ART cycles, patients usually have several embryos thus the best quality embryo can be transferred. In this study, we did not investigate the correlation between the conception rate and the quality of the transferred embryo, however, we did find a positive correlation with not only a higher number of transferred embryos but also with a higher number of received oocytes during the retrieval procedure. These correlations confirm the opinion that “every oocyte counts” as they could form more good quality embryos and increase pregnancy rates.

The day-2 serum progesterone has been estimated as a key hormone in predicting the ART outcome. Studies suggest that a high value of day-2 serum progesterone level was associated with a lower clinical pregnancy rate (Mahapatro and Radhakrishan, 2017). Likewise, other authors found the same result in IVF cycles using the GnRH antagonist protocol (Kolibianakis et al., 2004). Our research determined a positive statistical correlation between progesterone on day 2–3 and a successful conception. Since we only perform an IVF cycle when the progesterone is within normal levels, this correlation suggests that the fluctuations of progesterone under 1.0 ng/mL does not affect conception. We confirm that the progesterone measurement is an important clinical indicator for ensuring the chances of conception.

As mentioned above, infertility is a complex disorder influenced not only by one, but most commonly by multiple factors. In many cases the diagnosis does not provide useful insights, therefore, the causes of infertility may remain unknown. Difficulties in conceiving could also stem from the underlying molecular mechanisms in the endometrium or in other organs associated with reproduction (Gupta et al., 2014; Vázquez-Martínez et al., 2019; Fauque et al., 2020). Hoping that molecular analysis will give us useful insights, we investigated gene expression in the endometrial tissue and the follicular fluid cells between the two groups: in females unable to conceive (Group A) and in females who have successfully conceived (Group B). Aberrant gene expression patterns could be one of the causes of the endometrium dysfunction and, in turn, a contributing factor when it comes to difficulties conceiving (Garrido et al., 2002; Adamczyk et al., 2022b). The gene expression profiles obtained from RT-qPCR analysis demonstrated that Ten-eleven translocation (TET) genes *TET1* and *TET2* have a tendency to be upregulated in the endometrium of Group A. On the other hand, in the follicular fluid cells, these genes maintain a similar expression in both Group A and B. Proteins coded by these genes are associated with the changing DNA methylation pattern, more specifically, these proteins catalyze the DNA demethylation cascade (Ciesielski et al., 2017). Many studies suggest that the TET family plays a role in the endometriosis and endometrial cancer (Mahajan et al., 2020). One study investigated the *TET1* gene expression in the endometrium of the group of infertile endometriosis patients, infertile non-endometriosis patients, and in the group of patients without any reproductive disturbances (Adamczyk et al., 2022a). The study showed a significant decrease in the expression of *TET1* gene in the group of infertile endometriosis patients compared to fertile patients, however, the expression remained unchanged in idiopathic infertile patients. Moreover, lower *TET1* and *TET2* expression has been reported in the cases of endometrial cancer (Mahajan et al., 2020). Another gene of interest is a pro-apoptotic gene *BAK1* which is activated by p53 and BCL2. Our study found it to be significantly upregulated in the endometrium of Group A. This could suggest that apoptotic processes are induced by a greater degree in unexplained infertility patients than in fertile patients resulting in the stimulation of cell death. Most studies link BAK1 as well as BCL2 with the regulation of cancer. One of such studies by Witek et al. (2016) examined gene expression profiles in endometrial cancer and revealed that *BAK1* is overexpressed in grade 3 endometrial cancer which suggests that the tumor suppression system is still active and has yet to fulfil its role (Witek et al., 2016). There is evidence that augmented expression of a high mobility group AT-hook 2 (*HMGA2*) is associated with common benign mesenchymal tumors and rare aggressive cancers (Hammond and Sharpless, 2008). Moreover, HMGA2 plays a role in self-renewal and proliferation in neural stem cells since it was discovered that HMGA2 expression in mice decreases with age which in turn reduces the stem cell frequency and function (Nishino et al., 2008). Our research revealed a constant expression of *HMGA2* gene both in the endometrium and in the follicular fluid cells of both groups. As mentioned previously, many factors are possible contributors to infertility such as age, fertilization method, impaired quality of oocyte, hormonal imbalance. In addition to this list, the immune conditions may also lead to the reduced ability to conceive. A study by An et al. (2015) proposed that immunologic infertility could be caused by the upregulation of cytokines IL-2, IL-4, IL-6, IL-21, TNFα, IFNγ and IL-8 (An et al., 2015). Thirty infertile women were enrolled in this study and their blood was tested against mentioned cytokines. The analysis revealed a significant upregulation of these factors in the serums of infertility patients compared to healthy patients which led to a conclusion that the occurrence of immunologic infertility could be closely related to the changes in cytokine expression. Here we demonstrated the changes of the *IL-6* gene expression in the endometrium of infertile and fertile women. A significantly higher increase was recorded in the endometrium of infertile women (Group A), however, no significant changes were observed in the *IL-8* gene expression profile.

## Conclusion

A successful conception after ART in UI patients is more likely to occur in couples of younger parental ages with a good ovarian reserve and a higher number of transferred embryos. The duration of infertility can negatively affect conception rates, therefore, ART treatment should not be delayed in young UI couples. The gene expression analysis revealed significant changes in the endometrium regarding the genes associated with cell death (*BAK1*)*,* epigenetic DNA modification (*TET2*) and the immune response (*IL-6*) which were upregulated in females with the diagnosed unexplained infertility.

## Data Availability

The datasets presented in this study can be found in online repositories. The names of the repository/repositories and accession number(s) can be found in the article/Supplementary Material.
